# Gene networks activated by specific patterns of action potentials in dorsal root ganglia neurons

**DOI:** 10.1038/srep43765

**Published:** 2017-03-03

**Authors:** Philip R. Lee, Jonathan E. Cohen, Dumitru A. Iacobas, Sanda Iacobas, R. Douglas Fields

**Affiliations:** 1 Nervous System Development and Plasticity Section, the Eunice Kennedy Shriver National Institute of Child Health and Human Development (NICHD), NIH, Bethesda, MD, USA; 2 Department of Pathology, New York Medical College, Valhalla, NY, USA; 3 DP Purpura Department of Neuroscience, Albert Einstein College of Medicine, New York, NY, USA

## Abstract

Gene regulatory networks underlie the long-term changes in cell specification, growth of synaptic connections, and adaptation that occur throughout neonatal and postnatal life. Here we show that the transcriptional response in neurons is exquisitely sensitive to the temporal nature of action potential firing patterns. Neurons were electrically stimulated with the same number of action potentials, but with different inter-burst intervals. We found that these subtle alterations in the timing of action potential firing differentially regulates hundreds of genes, across many functional categories, through the activation or repression of distinct transcriptional networks. Our results demonstrate that the transcriptional response in neurons to environmental stimuli, coded in the pattern of action potential firing, can be very sensitive to the temporal nature of action potential delivery rather than the intensity of stimulation or the total number of action potentials delivered. These data identify temporal kinetics of action potential firing as critical components regulating intracellular signalling pathways and gene expression in neurons to extracellular cues during early development and throughout life.

Adaptation in the nervous system in response to external stimuli requires synthesis of new gene products in order to elicit long lasting changes in processes such as development, response to injury, learning, and memory^1^. Information in the environment is coded in the pattern of action-potential firing, therefore gene transcription must be regulated by the pattern of neuronal firing. Such transcriptional regulation is unique to neurons. The mechanisms that could be responsible for regulating gene expression by the temporal pattern of neuronal firing are largely unknown. Whether such regulation is limited to a few genes having special properties or extends to large numbers of genes by virtue of the dynamic response of intracellular signalling networks and cellular systems influencing transcription is unknown. How responsive the transcriptome is to very subtle alterations in patterns of action potentials remains an open, important question. There is very little data on the genomic response to the temporal features of action potential firing, where timing of action potential bursting is variable and the total delivery of action potentials over a given time period constant. Pharmacological manipulations, e.g., kainate^2^,^3^, pilocarpine^4^, NMDA^5^,^6^, KCl^7^, bicuculline^8^ and BDNF^7^ have been used to induce membrane depolarization followed by transcriptome analysis. These studies have detailed differential mRNA abundance, transcription factor regulation, regulatory sequences, chromatin occupancy of gene regulatory sites, and gene networks activated or repressed in neurons upon stimulation. However, a pharmacological approach induces stimulation that lacks temporal control of action potential firing patterns, which is critical during neural development and plasticity^6^,^8^,^9^. In contrast, altering the pattern of the same number of action potentials by using electrical stimulation produces a more naturalistic stimulus required to address the question of whether the abundance of RNAs and transcriptional networks are differentially sensitive to neuronal firing patterns.

We hypothesized that the temporal dynamics of action potentials would regulate the expression of hundreds of genes across broad functional categories^10^. In order to test the hypothesis that global mRNA abundance in neurons is dependent upon the specific pattern of action potential firing, mouse DRG neurons were electrically stimulated in cell culture with two distinct patterns of activation. These two patterns delivered the same total number of action potentials, but they were grouped into two different durations of firing at 10 Hz with different inter-burst intervals. These firing patterns are within the normal range of action potential firing in DRG neurons. Action potentials were delivered at a frequency of 10 Hz in 1.8 second bursts, repeated at 1 min intervals, or stimulated in 9 second bursts, repeated at 5 min intervals (termed 18/1 and 90/5 respectively). Stimulation was delivered for 2 or 5 hr and the abundance of 39,430 mRNA transcripts were compared with unstimulated neurons. DRG neurons were chosen for this study because they have no dendrites and do not form synapses (autapses) with other DRG neurons *in vivo* or in monoculture. Therefore action potential firing pattern could be controlled by electrical stimulation without complications of neural circuit activity mediated by synaptic connections and neurotransmitters.

Membrane depolarization and action-potential dependent Ca^2+^ influx by voltage-gated channels serve as the primary mechanism coupling neural activity and control of transcription, transport, and stability of mRNAs in neurons^11^,^12^. (In synaptic networks, NMDA receptor and other signalling pathways are also important in excitation-transcription coupling^13^,^14^, and local submembranous Ca^2+^ levels can be appreciable in the absence of measurable changes in cytoplasmic Ca^2+^). We previously reported the magnitude and temporal kinetics of intracellular Ca^2+^ responses evoked in DRG neurons by both of these stimulus patterns (18/1 and 90/5) and also found that levels of the immediate early gene (IEG) c*-fos* did not correlate with the magnitude of the evoked intracellular Ca^2+^ transient, but instead correlated inversely with the interval of time between bursts of action potentials^15^. Even a series of single action potentials can drive expression of c-*fos* if they are delivered at an appropriate interval (10 seconds)^16^. This suggested that gene expression is differentially regulated by differences in temporal dynamics of activation and inactivation of discrete/dissimilar sets of molecules within intracellular signalling networks that ultimately determine gene transcription or degradation of mRNA transcripts. In support of this hypothesis, MAPK phosphorylation, which is an important element in the signalling cascade linking membrane depolarization to gene transcription through intracellular Ca^2+^ fluxes, and phosphorylation of the transcription factor cAMP-response element binding protein (CREB), showed markedly different responses to these two patterns of stimulation^15^. MAPK is activated strongly by the 18/1 pattern of action potential firing, but not by the 90/5 pattern, whereas the transcription factor CREB is activated by both temporal patterns^15^. Thus expression level of specific genes that are influenced differentially by these two molecules should be selectively regulated by these two patterns of impulse firing. This hypothesis is tested here by genome-wide transcriptome analysis of all mRNAs. By extension, many other genes may be differentially regulated by temporal responses of different components in the intracellular signalling network beyond those regulated by CREB or MAPK.

Our results demonstrate that the abundance of hundreds of mRNAs in neurons across many functional gene categories can be modulated by the temporal pattern of neuronal firing. We identified functional gene categories and regulatory sequences in DNA that control activation and repression of these genes differentially in response to these two patterns of action potential firing. These genes include not only IEGs known to be responsive to action potential firing, but also categories of genes involved in neurite outgrowth, nervous system development, plasticity, and other neuronal functions.

## Results

### Temporal control of gene-regulatory networks by specific patterns of AP firing

**Figure 1:**
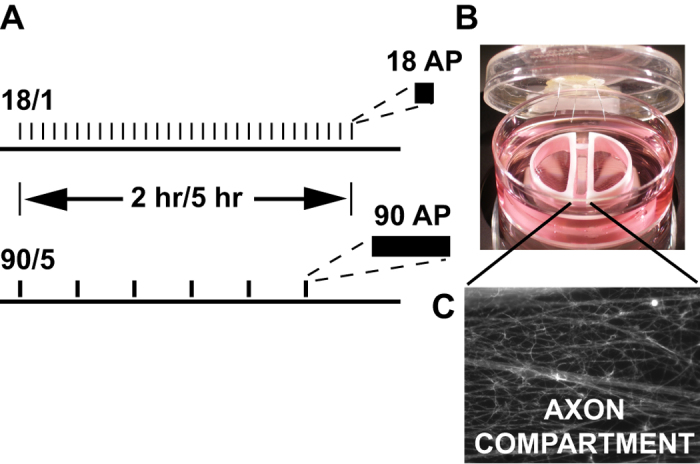
Stimulation patterns used in this study. (**A**) Shown are two patterns of electrical stimulation used throughout this study. For both 18/1 and 90/5 stimulation patters, an equal number of electrical pulses were delivered by bipolar stimulating electrodes across a Campenot chamber. 18/1 and 90/5 refer to 18 electrical stimuli delivered every minute (10 Hz) or 90 electrical stimuli (10 Hz) delivered once every 5 min for the duration of stimulation, respectively. In (**B** and **C**), is a representative image of a Campenot chamber used for long-term culture of DRG neurons showing axonal outgrowth (**C**) in the central compartment. Platinum electrodes deliver biphasic stimulation to both cell body compartments.

**Figure 2:**
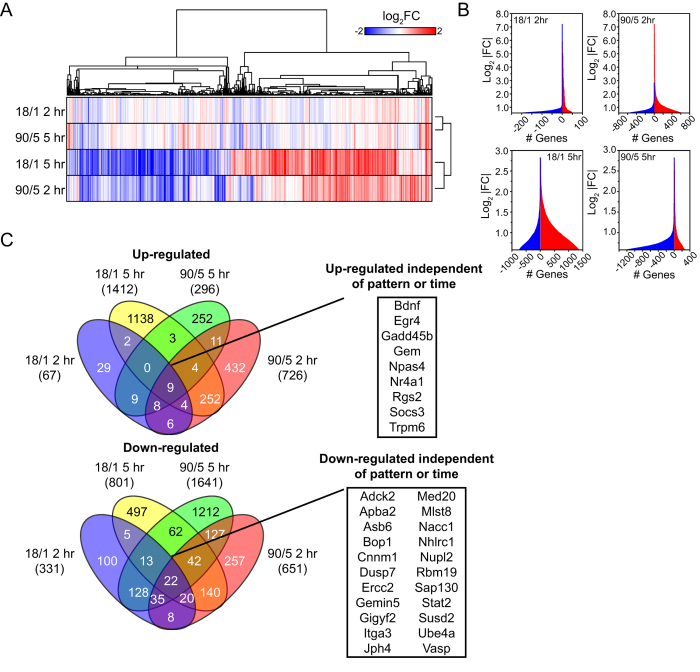
Action potential pattern dependent regulation of gene-regulatory networks. Neurons were electrically stimulated with two patterns, 18/1 or 90/5 for 2 hr and 5 hr. (**A**) Hierarchical clustering of log-normalized microarray data showing differential expression of many hundreds of genes in electrically stimulated DRG neurons. (**B**) Frequency distribution of up- (red) and down-regulated (blue) genes in DRG neurons. Each gene is represented along the x-axis and corresponding log-normalized expression value. Numbers of up- and down-regulated genes are unbalanced, favoring greater transcriptional activation at 5 hr for 18/1 stimulus pattern compared to greater initial activation at the 90/5 stimulus pattern at 2 hr and down-regulation by 5 hr. (**C**) Venn diagram showing numbers of up- and down-regulated genes at 2 hr and 5 hr for both patterns of electrical activation. Total numbers of regulated genes are given in parenthesis for each time, pattern, and direction of change. In total, 2,501 genes were significantly upregulated by electrical stimulation and 3,424 were significantly down-regulated by electrical stimulation. Of these, a small subset of genes was co-regulated by both patterns. Only 9 activity-regulated genes were co-upregulated independently of pattern or stimulus duration in contrast to 22 genes suppressed by patterned activity. N = 4 replicate experiments per condition.

We found that the majority of transcripts in DRG neurons were not regulated significantly by either pattern of electrical activity after 2 or 5 hrs of stimulation (86.1% of transcripts, 24, 213 out of 28, 131 unique IDs). We identified hundreds of transcripts that were regulated in a stimulus pattern-specific manner (Figs 1 and 2A). Significantly more transcripts were regulated after 5 hr stimulation than 2 hr of stimulation with the 18/1 firing pattern. In contrast, the 90/5 stimulation pattern regulated more genes at 2 hr than after 5 hr (Fig. 2B). Some transcripts were regulated similarly by both patterns of stimulation (Fig. 2B and C). The majority of transcripts that were up-regulated by the 90/5 stimulus pattern at 2 hr were subsequently down-regulated by 5 hr of stimulation. Of the 9 transcripts that were found to be up-regulated at both 2 hr and 5 hr stimulation at 18/1 and the 90/5 stimulus patterns, several are “core” activity-regulated genes that function as transcriptional regulators (*Gadd45b, Npas4, Egr4*, and *Nr4a1, Nur77*, and *Ngfib*), GTP-binding protein regulators (*Gem* and *Rgs2*) and growth factor signalling (*Bdnf, Soc3*). In contrast, none of the 22 transcripts downregulated in this stimulus/response category have been previously shown to be either activity-regulated or to have an established role in nervous system development. These very different gene expression profiles following electrical activity suggest that distinct gene regulatory networks are recruited by distinct action potential firing patterns (Fig. 2C). We therefore performed pathway analysis of gene-regulatory networks, signalling cascades, and gene ontologies that are recruited by distinct patterns of activity over time.

### Pathway analysis of gene networks activated by different action potential firing patterns

**Figure 3:**
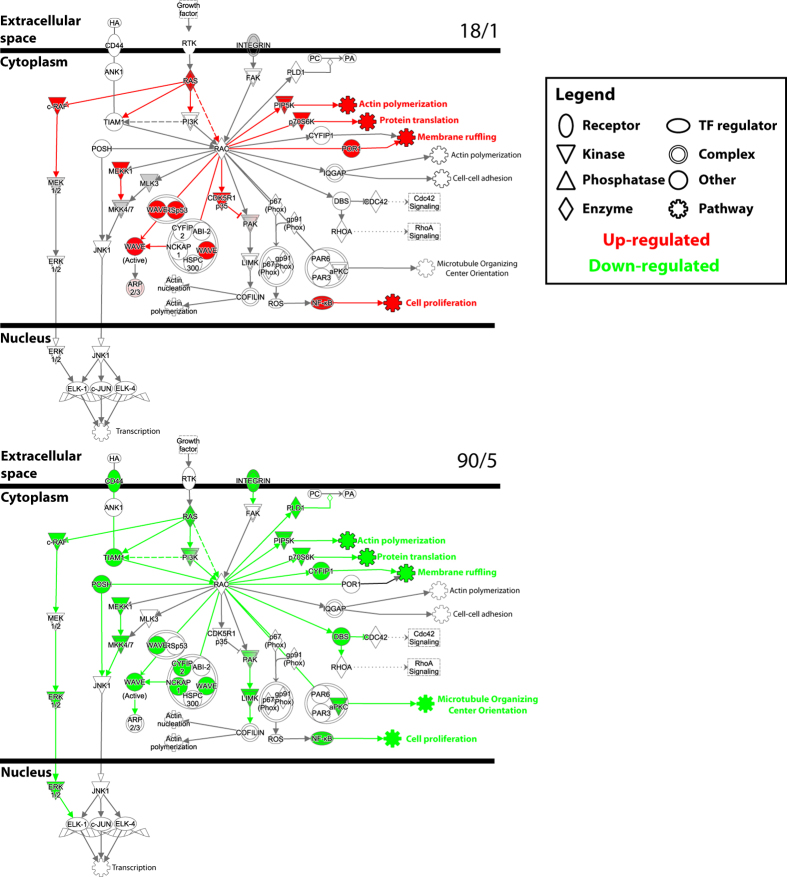
Opposing regulation of nerve growth factor and *Rac* signalling in DRG neurons. Pathway analysis of enriched canonical pathways identifies opposing regulation of *Rac* signalling by 18/1 and 90/5 patterns of stimulation. *Rac* signalling pathways were oriented to depict extracellular, cytoplasmic, and nuclear sites of action. Nodes in the pathway are up- (25, red) or down-regulated (11, green) depending on the regulation of target pathway genes. Shaded targets reflect multiple regulated components (e.g. MAPK pathway in 18/1 stimulation pattern). Multiple downstream pathways interacting through *Rac*, e.g., actin polymerization, translation and membrane ruffling, affect neurite outgrowth and dynamics.

We identified distinct gene networks that were activated by the two stimulus patterns after 5 hr of stimulation (Fig. 2, Tables 1 and 2). These included common disease and functional networks after the 18/1 (Table 1) and 90/5 (Table 2) stimulation patterns; e.g., neurological disease, cellular assembly and organization, and small molecule biochemistry. We have previously found that 18/1 and 90/5 stimulus patterns recruit Ca^2+^ and MAPK signalling pathways differently^15^,^16^; this was confirmed by analysis of canonical pathways enriched for both stimulation patterns. Pathways important in axonal growth and growth-cone signalling were oppositely regulated by the 18/1 and 90/5 stimulus patterns (Tables 3 and 4), e.g., the genes *Cdk5*-, *Rac*-, and *Ngf*-signalling networks. Pathways and gene networks important in axonal growth and development were oppositely regulated by the two distinct patterns of electrical stimulation (Fig. 3). Nerve growth factor (NGF) and cell adhesion (integrin) signalling pathways functioning through *Rac*-*Rho* GTP-binding proteins showed many differentially regulated transcripts (13 focus molecules up-regulated by the 18/1 stimulus and 25 down-regulated by the 90/5 stimulus). This suggests that genes in the pathways that are differentially activated by these two stimulus patterns may share transcription factor binding sites that are preferentially responsive to each of the two patterns of action potentials. We therefore analyzed conserved upstream regulatory elements of activity-regulated genes to identify over- and under-represented TFBS in the genes regulated by each stimulus pattern.

### Identification of transcription factor binding sites in genes differentially regulated by different action potential firing patterns

In search for TFBS enriched in genes regulated by specific action potential firing patterns, we analyzed transcriptome data by two techniques: an enhancer identification method through distant regulatory elements of co-regulated genes (DIRE)^17^ and by gene set enrichment analysis (GSEA)^18^. The sensitivity of DIRE was validated by analyzing transcriptome data where either CREB-regulated genes (526 genes), NF-κB regulated genes (403 genes), or MAPK-regulated genes (210 genes) were enriched (Fig. S2) (See methods for description of analysis and datasets for CREB, NF-κB, and MAPK). We confirmed the enrichment of several transcription-factor binding sites (TFBS) for CREB, ATF, VJUN, ATF3, and TAXCREB and to a lesser extent, NF-κB TFBS (NF-κB p65) in differentially regulated transcripts. As we expected, NF-κB target genes were enriched for NF-κB specific binding sites (p50, p65, and NF-κB) and Ets2^19^. In contrast, MAPK acts through multiple signalling pathways and may recruit many transcription factors. We identified a more diverse set of TFBS, prolactin receptor^20^, p53^21^,^22^, and TATA (despite being present in 25% of upstream regions), through analysis of down-regulated transcripts by targeted deletion of *Mapk* in embryonic DRG neurons^23^ consistent with converging action of several upstream signalling networks.

**Figure 4:**
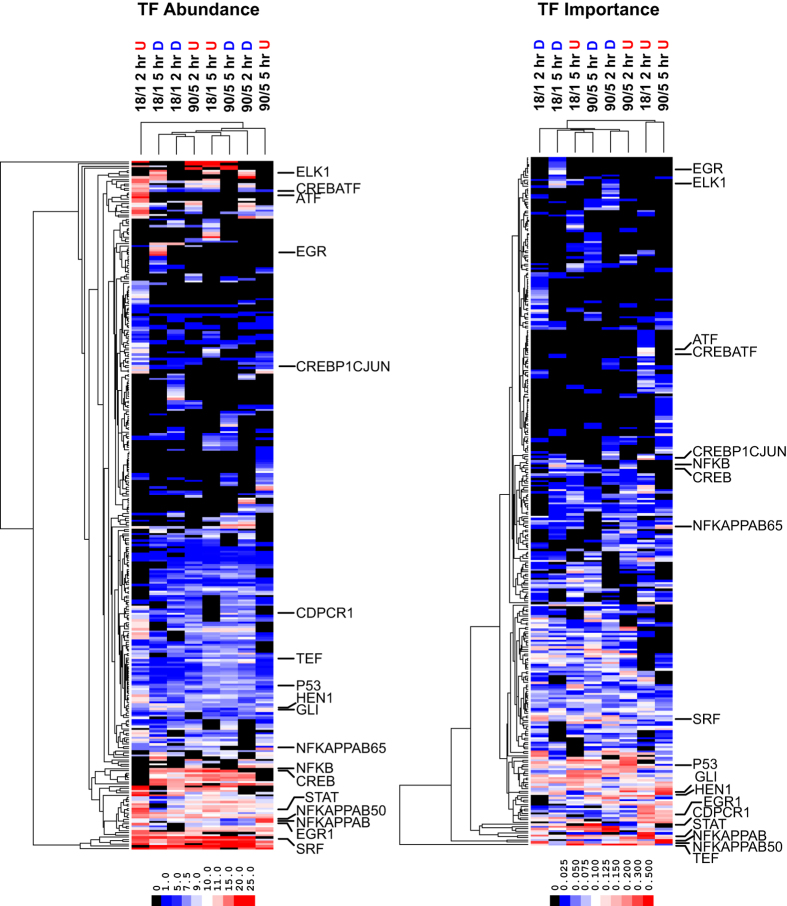
Enrichment of activity-regulated transcription factor binding sites. Hierarchical clustering of 311 TFBS by site and condition. Cis-regulatory elements identified through evolutionary conserved regions (ECRs), promoters, and 5′-UTRs are clustered by % occurrence within a gene set and by importance factor (TF abundance X TF weighting) for 18/1 and 90/5 stimulation patterns at 2 hr and 5 hr. Background gene sets (305 genes) were determined by filtering microarray data to identify genes not regulated by either pattern at 2 hr and 5 hr (fold-change < |1.4|), and used to calculate weighting for TF analysis. Enriched TFBS are annotated for both % abundance and importance.

**Figure 5:**
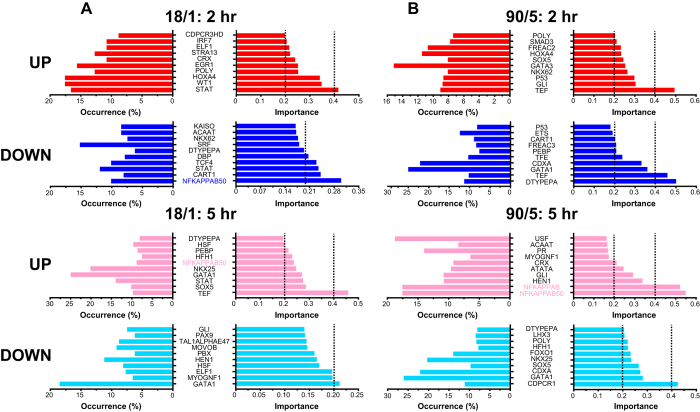
Top 10 candidate transcription factor binding sites enriched by stimulus pattern and duration. Top 10 candidate cis-regulatory elements are shown for genes activated by the 18/1 (**A**) and 90/5 (**B**) stimulation patterns after 2 hr and 5 hr. Importance factor scores >0.2 may be functionally significant as measured by enriched datasets for NF-κB and CREB binding site and downstream regulators of MAPK signalling in DRG neurons (Figure S2). Several candidate sites have high importance factor scores for multiple conditions, e.g. *Tef*. The TFBS used for this analysis are not differentiated by enhancer, repressor, or activation.

We applied DIRE analysis to identify upstream regulatory elements enriched in up- and down-regulated transcripts by 18/1 or 90/5 electrical stimulation patterns and performed hierarchical clustering by TFBS site and condition of the abundance and importance factor of 311 TFBS (Fig. 4). Of all the identified TFBS, 53 were present in one condition and 39 had variable abundance across all 8 conditions (Table S3). The most abundant TFBS present in all activity-regulated transcripts were MAF, Nkx2.5, STAT, and SRF. In order to discover sites that may be recruited in a pattern- and time-dependent manner, we applied a background set of non-regulated transcripts to scale TF abundance^24^. The identified sites may correspond to core, enhancer, or repressor elements. Putative enhancer sites would be expected to be over-represented in up-regulated transcripts; conversely repressor sites would be enriched in down-regulated transcripts. We discovered several top-scoring TFBS (top 10 by importance factor) through analysis of co-regulated genes (Fig. 5). Thyrotroph embryonic factor (*Tef*), a bZIP-family TF, forms heterodimers with other transcription factors in order to act as either an enhancer or repressor^25^ TEF sites were enriched in up- and down-regulated transcripts by 18/1 stimulatin (5 hr) and 90/5 stimulation (2 hr). STAT TF sites (JAK/STAT signalling, cytokines, regeneration) were selectively over-represented in transcripts up-regulated by 18/1 stimulation at 2 hr. NF-κB and NF-κB p50 enhancer binding sites were the most over-represented sites in transcripts up-regulated by 90/5 stimulation at 5 hr. Conversely, CCAAT-displacement protein CUT (*Cdpcr1*)^26^,^27^ and GATA1^28^,^29^,^30^, transcriptional repressors of developmental and synaptic genes, were most over-represented in down-regulated transcripts by the 90/5 stimulation pattern. NF-κB sites were significantly enriched when compared across transcripts (promoter and ECR) or TFBS (NF-κB, NF-κB p50, NF-κB p65) (Table 3).

Recognition sites for serum response factor (SRF) and CREB were significantly enriched by 18/1 stimulation at 2 hr (FDR < 0.05) (Table S2) when transcriptome data was independently analyzed by gene set enrichment analysis (GSEA). SRF and CREB sites were only observed after 5 hr stimulation by the 90/5 pattern. As we observed with DIRE, NF-κB sites (NF-κB, NF-κB p65), were only significantly enriched by the 90/5 stimulus at 5 hr. Two TFBS were over-represented across all conditions, FREAC3 (forkhead transcription factor site, FOXC1) and MYOGNF1 (myogenin/nuclear factor 1). These findings were consistent with recruitment of distinct transcriptional networks by patterned action potential firing activity.

**Figure 6:**
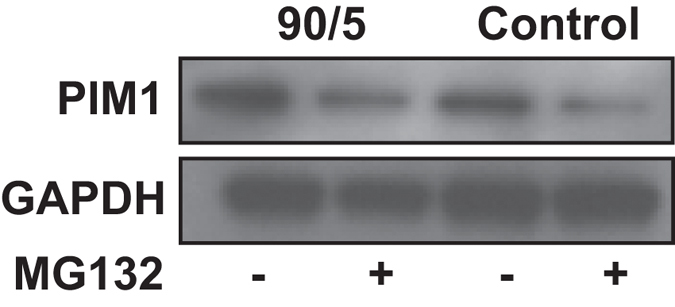
NF-κB target gene PIM1 is up-regulated by patterned action potential activity. Protein expression levels of PIM1 following 90/5 stimulation for 5 hr were measured by immunoblotting. 10 μM MG132, a general proteasome and NF-κB inhibitor, was pre-incubated for 2 hr prior to stimulation. MG132 treatment reduced basal levels of PIM1 expression. GAPDH was used as a loading control.

To demonstrate specific activation of the NF-kappa transcriptional network by 90/5 electrical stimulation, we identified transcripts through DIRE and GSEA that were enriched by having multiple NF-κB sites. We performed RT-PCR on 3 genes that had enriched NF-κB TFBS: BCL6 Co-Repressor (BCOR), proto-oncogene serine/threonine-protein kinase (PIM1), and NF κB Inhibitor Epsilon (NFKBIE). All 3 transcripts were significantly increased by 5 hr of the 90/5 stimulation pattern and were not changed by the 18/1 stimulus pattern (Table S1). We further analyzed the protein expression levels of PIM1 by immunoblotting, which was consistent with the mRNA data. The proto-oncogene serine/threonine-protein kinase, Pim1, controls NF-κB activation through stabilization of RelA-p65 by phosphorylation^31^. Pim1 was expressed in DRG neurons (Fig. 6) and pre-treatment with the general proteasome and NF-κB inhibitor MG132 (10 μM) reduced both basal and activity-induced expression of Pim1.

## Discussion

These studies demonstrate that a critical factor in the regulation of gene expression in neurons is the temporal nature of action potential firing. We have identified large groups of genes whose mRNAs, across many functional categories, can be differentially regulated by both the temporal dynamics of action potential firing and total length of stimulus time. Thus in neurons, the transcriptome in general, and not simply a specialized set of genes, is highly regulated by the state of neural impulse activity. The implications of the broad categories of genes being regulated by the pattern of neural impulse firing is that the effect of other signalling molecules, growth factors, etc., on neurons will vary greatly depending on the state of activity of the neuron. For example, as discussed below, factors influencing gene expression that stimulate neurite outgrowth will have different responses depending on the state of impulse activity in the neuron through the broad activity-dependent changes in the transcriptome. Likewise, by regulating neural activity, neurite outgrowth and other neuronal responses could be regulated independently of signalling molecules or drug treatments. It may be possible to exploit activity-dependent regulation of the transcriptome therapeutically, for example in promoting recovery from neural injury.

We have classified these genes by the occurrence of TFBS in the promoter regions of genes specifically activated by action potential pattern and/or time of stimulation. These analyses reveal that many genes containing binding sites for well-known activity-regulated transcription factors, such as CREB and SRF are regulated without regard to the two different action potential firing patterns (18/1 and 90/5) after 2 and 5 hr of stimulation. However, we have found that a subset of TFBS, such as NF-κB, are overrepresented in genes whose abundance was regulated differently by the two action potential firing patterns tested. This strongly suggests that the activation and repression of gene networks and subsequent control of gene expression in neurons, is subject to temporal control by the timing of intracellular Ca^2+^ transients, rather than the overall concentration of Ca^2+^ within the cell.

We have shown that two different patterns of electrical stimulation that produced the same number of action potentials regulate distinct gene regulatory networks. Surprisingly, very few transcripts were regulated in a time- and pattern-independent manner (Fig. 2C inset). 8 of the 9 up-regulated transcripts have been previously described as IEGs, such as the transcription factors *Egr4* and *Npas4*, whereas *Bdnf* is one of the most studied IEG growth factors^32^. The TRP-M channel, *Trpm6* (transient receptor potential cation channel subfamily M member 6), which was up regulated in all stimulus conditions, has an important role in magnesium homeostasis^33^, but a defined function in sensory neurons has not been shown. Historically, less interest has been placed on identifying genes or networks that are down-regulated by neural activity. The results of the present experiments revealed thousands of genes that are down-regulated in a pattern- and time-dependent manner. However, only 22 transcripts (Fig. 2C) were strongly down-regulated, independent of stimulus pattern. These genes have no shared network or function in common. Many of these genes have a role in transcriptional and translational regulation. However, by TFBS analysis, several predicted TFBS sites associated with repressor function were enriched in these genes; TAL1, Nkx2.5, ZNF219^34^ and NERF-ELF2 can function as repressors^35^,^36^. This is suggestive of transcriptional pathways that require derepression in order to facilitate the expression of activity-regulated genes, a less studied part of the neuronal activity-driven transcriptome. Further work is required to elucidate a role for the repressors which can perhaps lead to a common pathway or pathways of genes required to be downregulated during neuronal plasticity.

Analysis of gene networks using Ingenuity Pathway Analysis (IPA) demonstrates that the temporal dynamics of action potential firing can regulate pathways involved in growth of neurons. Figure 3 shows that the 2 patterns of stimulation used in this study have opposite effects on the abundance of many mRNAs involved in neurite outgrowth through the Rac-GTPase signalling pathway. The 18/1 stimulus pattern appears to be growth promoting whilst the 90/5 stimulus pattern downregulates many of the same genes and pathway components. Many of these same genes have been shown to regulate neurite outgrowth in cortical neurons^37^. It has been shown previously that growth cone migration and neurite outgrowth are regulated by patterned activity in DRG neurons^38^.

Our findings support the hypothesis that transcriptional control of gene expression by the temporal pattern of action potential firing occurs through recruitment of distinct networks (co-regulated sites/heterodimer binding sites) of transcription factors and binding sites. Furthermore, these results show that genes with predicted NF-κB TFBS are over-represented in genes regulated by the 90/5 pattern of stimulation (Fig. 4, Table 5, Table S2). Two independent analysis methods (DIRE and GSEA) identified sets of sites in genes that were regulated by both stimulation patterns (18/1 and 90/5) and time (2 hr and 5 hr). We have classified many distinct transcription factor pathways and their occurrence in genes regulated by patterned action potential firing. Binding sites for SRF were one of the most abundant of identified TFBS by DIRE (14 ± 1%) and enriched following 2 hr stimulation with the 18/1 pattern by GSEA (4 predicted TFBS, Table S2). More importantly, several CREB sites (ATF, CREBATF, and TAXCREB) were specifically enriched in the 18/1 pattern at 2 hr (Fig. 4 and Table S2). These finding suggest that well characterized activity-dependent transcriptional networks, e.g. SRF alone are not sensitive to patterns of action potentials and require interactions through complexes of factors, e.g. CREB.

We identified thyrotroph embryonic factor (*Tef*) sites that were over-represented in up- and down-regulated transcripts following 2 hr stimulation in the 90/5 pattern. *Tef* has previously been shown to control circadian rhythm-mediated gene expression. *Tef* is expressed in DRG neurons and itself downregulated by the 90/5 stimulus pattern at 5 hr. TEF, like other TFs, forms heterodimers with other factors to function as enhancer or repressor complexes^39^,^40^,^41^. GATA1 TFBS were over-represented in down-regulated transcripts (Figs 4 and 5), may fine-tune TEF to form pattern-specific repressor complexes. However, transcript levels of several GATA family members is at very low levels (*GATA 1–4*), *GATA5* and *GATA6*, and *GATA-d1, 2a (p66α*), *2b (p66β*) are expressed at moderate to high levels. Despite the variable expression of *GATA* isoforms in unstimulated DRG neurons, transcript levels of *GATA* isoforms were globally repressed by patterned activity, e.g. *GATA5* by 90/5 at 2 hr (−2.3 fold, p < 0.005) and 5 hr (−1.6 fold, p < 0.001). This regulation may occur through conserved upstream regions of GATA genes. Prolactin receptor (PR) TFBS were enriched in ERK-MAPK regulated transcripts (Fig. S2); PR sites were modestly enriched in up-regulated transcripts at 2 hr and 5 hr, irrespective of pattern.

Our findings predict that the NF-κB transcriptional network and other cis-acting elements, are sensitive to the pattern of action potential delivery. Given the importance of NF-κB in Ca^2+^-dependent signalling and transcriptional regulation in many cells types including neurons^42^ it is perhaps surprising that there is little information regarding genomic and network regulation involving the NF-κB family of transcription factors in the nervous system. Recently, Ca^2+^ spike duration has been shown to regulate NF-κB transcriptional activity in epithelial cells^43^. Electrical stimulation of the hind paw induces translocation and phosphorylation of NF-κB in small to medium DRG neurons^44^, supporting these findings. Furthermore, NF-κB activity affects neurite outgrowth in cultured DRG neurons^45^. Therefore, the NF-κB family of transcription factors could potentially play a key role in stimulus driven Ca^2+^ -dependent transcriptional regulation in sensory neurons.

Many other mechanisms can influence gene expression in neurons, such as regulation through epigenetic mechanisms, control of mRNA and protein stability and localization of mRNAs and proteins. However, we have demonstrated that action potential firing patterns can differentially recruit transcriptional networks in neurons leading to regulation of hundreds of transcripts.

In summary these results identify the temporal kinetics of action potentials, such as the duration of bursts of action potentials and the interval of time between repeated bursts, as key drivers in regulating gene expression in neurons through the recruitment of specific transcriptional networks to regulate hundreds of genes across all functional gene categories. The data presented here provides a large dataset on mRNA transcripts in neurons that are regulated in abundance by these different patterns of action potential firing. These data should be a valuable resource for many other investigations. The findings greatly increase our understanding of adaptive global gene regulation in response to environmental stimuli and demonstrate that gene expression in neurons is very sensitive to timing of action potential bursting, and that the activation of transcription factor networks is dependent upon the timing of action potential firing independent of the frequency of firing or the total number of action potentials delivered.

## Methods

### Primary neuronal cell culture

All experiments were conducted in accordance with animal study protocols approved by the NICHD Animal Care and Use Committee. For DRG cell culture, multi-compartment Campenot chambers made of Telfon, were affixed by vacuum grease to collagen-coated 35 mm cell culture dishes as previously described^46^,^47^. Neurons were dissociated from spinal cords of 13.5-day old mouse embryos and plated at a density of 0.5 × 10^6^ cells per ml into each side compartment in Eagle’s MEM containing 5% horse serum and 100 ng/ml nerve growth factor. Non-neuronal cell proliferation was inhibited by treatment with 13 μg/ml fluoro-2-deoxyurindine (Sigma, St. Louis, MO) by one day following plating for 5 days. Cultures were subsequently used for experiments 3–4 weeks after which axons extend from the side compartments into the central compartment and could be electrically stimulated.

### Electrical stimulation of DRG neurons

Prior to an experiment, DRG cultures were visually inspected for acceptable outgrowth under the Teflon barrier and medium was exchanged for medium lacking NGF and sera, stimulating lids were replaced and cultures were left overnight. The following day, DRG cultures were stimulated through platinum electrodes in contact with medium on opposite sides of the barrier. Stimulation parameters and responses to stimulation have been reported previously^48^. For this study, either 18 actions potentials every min (18/1) or 90 action potentials every 5 min (90/5) was delivered by a 6 V, 0.2 ms biphasic pulse for either 2 hr or 5 hr. Control (unstimulated) experiments were performed by handling cultures in an identical manner (from the same dissection) as experimental (stimulated) cultures, including exchanging medium and fitting stimulation electrodes, however these culture dishes were not attached to the stimulation apparatus. Unstimulated controls were prepared on the same day as stimulated cultures and RNA was prepared at the same time in both cases.

### Immunoblotting

Total protein was isolated from DRG cultures using M-PER mammalian protein isolation reagent (Thermo Fisher Scientific, Waltham, MA) supplemented with HALT^TM^ protease and phosphatase inhibitors cocktail (Thermo Fisher Scientific, Waltham, MA). Total protein was resolved by SDS-PAGE on 4–12% NuPAGE Bis-Tris gels (Life Technologies, Carlsbad, CA), transferred to PVDF membranes (Immobilon-P, Millipore, Bedford, MA) and blocked in TBS (10 Mm Tris-Cl, pH 7.5, 0.9% NaCl) containing 0.1% (v/v) Triton X-100 (TBS-T) and 5% (w/v) nonfat dry milk or 5% (w/v) bovine serum albumin for 2 hr at room temperature or overnight at 4 °C. Membranes were probed with appropriate antibodies in TBS-T and 5% BSA overnight at 4 °C or 4 hr at room temperature with shaking. Primary antibodies were visualized with HRP-conjugated secondary antibodies (Amersham Pharmacia Biotech, Piscataway, NJ) at 1:4000 dilution and enhanced chemiluminescence. Primary antibodies used for immunoblotting: GAPDH (Encor, Gainesville, FL) used at 1:2000 dilution and PIM1 (Cell Signaling, Danvers, MA) used at 1:1000 dilution.

### Semi-quantitative RT-PCR

Total RNA (2 μg) was extracted from unstimulated and stimulated DRG cultures after 5 hr using TRIzol (Invitrogen, Carlsbad, CA) and reverse transcribed using Superscript II (Invitrogen, Carlsbad, CA) as previously described^49^. Semi quantitative PCR was carried out using a Roche Lightcycler using the FastStart DNA Master SYBR Green PCR reaction mix (Roche Diagnostics, Indianapolis, IN) according to manufacturer’s protocol. Data were analysed by the 2(−ΔC (T)) method with respect to unstimulated cultures.

The following primer sequences were used:

GAPDH FP: AATGCATCCTGCACCACCAAC

GAPDH RP: TGGATGCAGGGATGATGTTCTG

NPAS4 FP: TGGCATATTGTCATCAAGACGTG

NPAS4 RP: TTGAAATGGAAACTGGGATCG

EGR1 FP: AAGCACAGGAGGGAAGAGATG

EGR1 RP: TGAAGTCAAAGGGAACAGGAC

NFKBIE FP: AGAGTGAGGAGGAAGAATATG

NFKBIE RP: TTCTCCTTCTTCGTGCATGAC

PIM1 FP: CAACTCATTCCAGACTCCAGG

PIM1 RP: TGACTAAAGTTCCATCGACAGG

BCOR FP: ATGGGTGTATTGTGTGGTGAGG

BCOR RP: ACATACCACTGGATCACGTCC.

### Microarray and bioinformatic analysis

Total RNA was harvested immediately after 2 or 5 hr stimulation and processed as previously described^49^. Each control and experimental condition was the result of 5 pooled cultures. All control and experimental manipulations were performed over a short time to minimize the effects of tissue culture variation on gene expression levels. 825 ng of differently (Cy3/Cy5) labeled RNAs from two biological replicas were co-hybridized 17 h over night at 65 °C with each array of 4 × 44k Agilent 60mer G2519F mouse chips (“multiple yellow strategy”^50^). Chips were scanned with an Agilent G2539A dual laser scanner at 5 μm pixel size/20-bit and raw data extracted with Agilent Feature Extraction software vs 11.1.1. All spots affected by local corruption or with foreground fluorescence less than twice the background in any of the 20 samples were disregarded. Data were normalized by an iterative method alternating intra- and inter-array normalization until the overall maximum error of estimate became less than 5%. Supplementary Figure 1 shows the Pearson correlation coefficients between the gene expression of all 6 sample pairs that can be formed in each condition. The high correlations prove the robustness of the data.

### Expression regulation

A gene was considered as significantly regulated in neurons subjected to α (=18/1 for 2 hr, 18/1 for 5 hr, 90/5 for 2 hr, 90/5 for 5 hr) pattern of stimulation with respect to non-stimulated (X) neurons if:


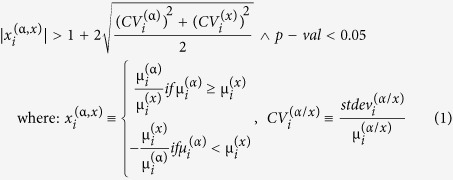


The composite criterion (1) requires that the absolute expression ratio exceeds the pooled coefficient of variability for the stimulated (pattern α) and non-stimulated neurons. The fold change cut-off was computed separately for each transcript and incorporates both biological variability and technical noise. This condition replaces the arbitrary 1.5 fold change cut-off that may be too stringent for very stably expressed genes (close values in all four replicates) or too relaxed for highly unstably expressed genes^50^. The p-values of the heteroscedastic *t-*test were computed with a Bonferroni type correction applied to the groups of spots probing the same transcript^51^.

A gene was considered as turned on by a particular stimulation pattern if it was not quantifiable (i.e. foreground fluorescence less than twice the background) in any of the unstimulated neuronal samples but quantifiable in all stimulated samples with that pattern. Turned off genes satisfied the opposite criterion i.e. expressed in all not stimulated samples but not expressed in any of the stimulated ones. Raw and normalized gene expression data were deposited into GEO and are publicly available under accession number GSE84872.

### Bioinformatic analysis

Hierarchical clustering and frequency distribution of Agilent microarray expression data was performed using Cluster 3 (http://bonsai.hgc.jp/~mdehoon/software/cluster/)^52^ and JTree View (http://jtreeview.sourceforge.net/) on Log2 normalized data. Expression data was filtered according to direction (up- and down-regulated), pattern (18/1 and 90/5), and time (2 hr and 5 hr) where p < 0.05 and fold-change >|±1.4|. Gene ontology (GO), network, and pathway analyses was performed using pathway analysis software (Ingenuity Systems, Redwood City, CA). Four way Venn diagrams were constructed using the Java applet Venny, http://bioinfogp.cnb.csic.es/tools/venny/index.html.

Prediction of TFBS was performed using either distant regulatory elements of co-regulated genes (DIRE) or gene set enrichment analysis (GSEA). Sensitivity of DIRE in predicting TFBS enrichment was validated by analysis of gene sets corresponding to (**a**) VP16 constitutively active CREB (526 transcripts)^9^, (**b**) NF-κB regulated transcripts (403 transcripts) (http://www.bu.edu/nf-kb/gene-resources/target-genes/), and (**c**) sensory neuron specific ERK1/2 conditional knockout (210)^23^. DIRE accurately predicted the presence of ATF/CREB, NF-κB, and downstream MAPK TFBS (Fig. S2). For DIRE analysis of DRG transcriptome data, a background set of genes was identified as not regulated (fold-change < |1.1|) by either stimulation pattern (18/1 and 90/5) and time (2 hr and 5 hr). Gene symbols for co-regulated genes and background sets were uploaded to DIRE where the target elements corresponded to the Top 3 evolutionary conserved regions (ECR) and the promoter ECRs; results were reported as % abundance and importance factor. Hierarchical clustering of TFBS was performed as described for Agilent microarray data.

GSEA of gene expression data was performed with BROAD institute stand-alone application against the Molecular Signature Database (MSigDB). Microarray data (4 replicates per condition) was minimally processed (quantile normalized data of 30,564 entries collapsed to 8,546 entries and expression levels ± 1 S.D. of background). Expression values in each condition were analyzed as control vs. treatment (pattern and time) against a MSigDB comprising 836 gene sets (221 miRs and 615 TFs). MSigDB are pre-computed set of genes with promoter regions ± 2 kb of the TSS containing TFBS of interest. For identified signatures with a false discovery rate (FDR) of 0.25 or less, normalized enrichment score (NES) and FDR are given; in all cases local p-values < 0.05.

## Additional Information

**How to cite this article**: Lee, P. R. *et al*. Gene networks activated by specific patterns of action potentials in dorsal root ganglia neurons. *Sci. Rep.* **7**, 43765; doi: 10.1038/srep43765 (2017).

**Publisher's note:** Springer Nature remains neutral with regard to jurisdictional claims in published maps and institutional affiliations.

## Supplementary Material

Supplementary Material

Supplementary Dataset 1

## Figures and Tables

**Table 1:** Enriched gene networks recruited by 18/1 patterned action potential stimulation for 5 hr.

Network	Score	Top Disease and Function	Focus Molecules
1	38	Behavior, Nervous System Development and Function, Cellular Assembly and Organization	*AKTIP, ALAS1, CALU, CNOT10, CPT2, CRY2, DDX56, ECD, EHD2, FAM127C, GIGYF2, HDLBP, HOOK1, IGF1R, KIF21B, MRPL39, MRPS24, MRPS9, NUFIP1, PAN3, PDGF* (family), *PER1, PER2, PFDN2, PIH1D1, POLR3A, PRKCDBP, RPAP3, RQCD1, TELO2, TNRC6C, TRIM3, UBOX5, USP2, ZNF598*
2	38	Small Molecule Biochemistry, Nutritional Disease, Psychological Disorders	*ANKIB1, CEP250, CIZ1, CNR1, CUL7, DAP3, DDX21, DGCR14, EPB41L3, ERRFI1, FBL, GALNT18, GBP4, GCH1, GPCPD1, GPD2, HAO1, HSPA9, LCN12, MICALL1, MMEL1, MTMR1, NSUN4, NUP160, PABPC4, PACSIN3, PRMT3, PSPC1, RAB35, SLC25A13, SPICE1, SRSF10, TUFM, ZSWIM7*, methyltransferase
3	38	Cellular Assembly and Organization, Cellular Compromise, Dermatological Diseases and Conditions	*AIFM3, ARL2, ATAD1, ATPase, CARNS1, CCAR1, CUEDC1, DDX3X, DDX54, DEDD2, DMTN, DPP3, ELMOD2, ETNK2, FAM89B, GRIP1, LOR, MFSD2A, NEDD4L, NR2F2, NR3C1, PEX6, PIM3, PRRG1, RAD54L2, RNF10, SMARCA4, TCF19, TMEM55A, TULP3, UBTD1, WDR19, WRNIP1, ZNF462, ZNF704*
4	38	Hereditary Disorder, Neurological Disease, Psychological Disorders	*ADNP2, AGPAT3, AOC2, ATP5J2-PTCD1, BIN3, BMS1, CADM3, CADM4, CCDC12, CREB3, CRYZL1, FBXL4, GOLPH3L, HEBP2, HSD17B7, Histone h3, KAT7, KLHDC10, MFSD5, NAB1, RBM6, RNASEH2A, SCRT1, SDSL, SHMT2, SLC29A2, SLC35C2, SLC41A3, SNX14, SSRP1, SYNGR1, TMEM140, VKORC1, XRCC6, ZNF239*
5	36	Cellular Assembly and Organization, DNA Replication, Recombination, and Repair, Developmental Disorder	*ABTB1, ANKRD32, APEH, ASB6, ASF1A, ASF1B, AUP1, BPTF, BRF2, CAPRIN2, CBR1, CHRAC1, CSDC2, DIP2B, ERCC8, Ewsr1, FRZB, Frizzled, HIST1H3A, HOXD1, ISL2, KDM4C, LDB1, OTUD4, PLC, POLE3, PREB, RNF38, SMARCA1, SMARCA5, SNAPC4, SSBP4, UBA5, UBN1, XAB2*

Network discovery by IPA of enriched disease and function. Network score are listed for 5 top-scoring networks, encompassing processes important in nervous system development, function, and disease.

**Table 2:** Enriched gene networks recruited by 90/5 action potential patterned stimulation for 5 hr.

Network	Score	Top Disease and Function	Focus Molecules
1	39	Cell Morphology, Connective Tissue Development and Function, Tissue Morphology	*ABCC5, ABCF1, ATM/ATR, CDKAL1, CTU1, DDX52, DPH2, FAM120B, GPANK1, GULP1, HLCS, KIAA1161, MMS19, MON2, MSL2, MXD3, MYO18A, NCAPD3, NCAPH2, NKAP, ORAOV1, PHLPP1, PRIM2, RBM39, SLC12A2, SLC30A1, SLC38A2, SLC4A7, SLC7A1, SMC2, SMC3, SNX27, SRC*
2	38	Neurological Disease, Small Molecule Biochemistry, Developmental Disorder	*ACAD10, APP, ATCAY, CHCHD6, DPY19L3, FRMD8, GSPT2, HBS1L, IMP4, ISOC2, KCTD18, LETM2, LHFPL2, MAB21L2, NARF, ORMDL1, PATL1, PHACTR4, PLEKHO2, PUS7L, RAB39A, RCBTB1, RNF24, RP9, Rbfox3, SLC25A38, SLC41A3, TBC1D25, TCP11L2, UGGT2, ULK4, VAT1L, ZC2HC1A, ZNF322, ZNF35*
3	37	Post-Translational Modification, Cell Morphology, Cellular Assembly and Organization	*AFAP1, ATE1, Ank2, CEP170, DUB, FTO, HERC2, KCNC4, KIF3A, KIF3B, KIF3C, MED20, NPR2, PAN2, PCYT1A, Par6, Pkc(s), RNF168, SLC18A3, SLC6A9, TRIM46, USP1, USP10, USP12, USP28, USP29, USP30, USP32, USP38, USP40, USP42, USP54, WDR20, WDR62, ZRANB1*
4	37	Cancer, Gastrointestinal Disease, Organismal Injury and Abnormalities	*ABR, ADIPOR2, AGL, APPL1, CENPC, DARS2, DPYSL5, EIF5B, ERBB2IP, GAPVD1, INSM1, KCNA5, KCNS3, KCTD5, KIF26B, MINK1, MPP3, NELL1, NHLRC1, NIT1, NSL1, OCRL, PKNOX1, PRKAB2, RAB11FIP5, RPAP1, Rab11, Rab5, Rac, SLTM, TPCN2, TROVE2, ZBTB3, ZFYVE9, ZNF202*
5	37	Cell-To-Cell Signalling and Interaction, Cellular Movement, Cellular Development	Alpha catenin, *BAG2, BSN, CCDC117, CDH2, CDH4, CDH8, CEP68, CTBP1, CTNND1, Cadherin, DEF6, ERC2, FOXJ3, HDAC11, IQSEC1, KCTD6, LTN1, MLF2, NUP85, OGT, PHF20, RBBP5, RBM22, RNF111, S1PR1, SATB1, SSPN*, Spectrin, *TJP1, TMEM117, TRIM26, TRMT2A, ZEB2, ZKSCAN8*

Network discovery by IPA of enriched disease and function. Network score are listed for 5 top-scoring networks, encompassing processes important in cellular development, morphology, and disease.

**Table 3:** Enriched canonical pathways by 18/1 patterned stimulation for 2 hr and 5 hr.

Pathway	# mol	Z-score	p	Molecules
**2** **hr**				
IGF-1 Signalling	7	−1.13	4.41e-3	*FOS, CSNK2A2, SOCS3, SHC1, PRKAG2, STAT3, SOCS5*
ERK/MAPK Signalling	8	−2.12	3.76e-2	*FOS, SHC1, ITGA3, PRKAG2, DUSP4, PAK7, RPS6KA1, STAT3*
Tec Kinase Signalling	7	−1.63	4.62e-2	*FOS, ITGA3, GNB2, STAT2, PAK7, STAT3, ITK*
Axonal Guidance Signalling	15	—	3.80e-2	*RGS3, SLIT1, BDNF, NFATC3, C9orf3, EPHA1, WNT9A, PLCH2, LIMK1, SHC1, ITGA3, PRKAG2, GNB2, PAK7, VASP*
**5** **hr**				
CDK5 Signalling	20	−0.47	1.07e-2	*ITGB1, PPP1R14C, RAF1, BDNF, PPP2R5D, PPP2R5B, ADCY6, MAPK12, CACNA1A, CDK5R1, ADCY9, PPP1R3D, ITGA3, PPP1R12A, MAP2K2, CAPN1, PRKAG2, MRAS, PPP2R5E, MAPK7*
Death Receptor Signalling	19	2.07	9.45e-3	*MAP2K4, GAS2, RELA, MAP2K7, PARP8, TNKS2, FAS, ACIN1, DAXX, TRADD, TRAF2, CASP9, CRADD, TNFSF12, BID, HTRA2, CHUK, NFKBIB, TNFRSF10A*
Rac signalling	20	1.34	2.29e-2	*MAP2K4, ITGB1, RELA, RPS6KB1, RAF1, MAP2K7, MAP3K11, ARPC5L, MAP3K1, PIKFYVE, PIP4K2B, WASF1, CDK5R1, PAK1, ITGA3, MAP2K2, BAIAP2, MRAS, PIP5KL1, PAK7*
ERK5 signalling	13	1.73	3.76e-2	*IL6ST, RPS6KB1, BAD, CREB3, CREBBP, MYC, RPS6KA6, MEF2D, NTRK1, MRAS, FOSL1, MAPK7, RPS6KA1*
NGF signalling	19	1.41	4.20e.2	*MAP2K4, GAS2, RELA, MAP2K7, PARP8, TNKS2, FAS, ACIN1, DAXX, TRADD, TRAF2, CASP9, CRADD, TNFSF12, BID, HTRA2, CHUK, NFKBIB, TNFRSF10A*

IPA analysis was performed on up- and down-regulated transcripts (>|1.4|-fold and p < 0.05) as described in Fig. 2 and mapped to cellular and molecular signalling pathways. Z-score represents the predicted pathway activation (+) or inhibition. Significance values were calculated by a Fisher’s exact test (right-tailed) to determine the probability that a pathway was attributable to chance alone. Enriched pathways are shown, demonstrating opposing activation (18/1) and inhibition (90/5) for Nerve Growth Factor (NGF) and Rac signalling (Fig. 3) important for neurite outgrowth.

**Table 4:** Enriched canonical pathways by 90/5 patterned stimulation for 2 hr and 5 hr.

Pathway	# mol	Z-score	p	Molecules
**2** **hr**				
p53	14	1.27	9.23e-3	*GADD45B, JMY, TOPBP1, PIK3R1, BAX, TP53BP2, CCNG1, PIK3R3, BBC3, PPP1R13B, SIRT1, ADGRB1, GSK3B, TNFRSF10A*
Tec Kinase	19	−0.258	1.72e-2	*TNFRSF21, STAT5A, PIK3R1, STAT3, GNG7, PIK3R3, FOS, GNAI3, ITGA3, LCK, TNFSF12, PAK2, GNAO1, MAPK10, STAT2, RHOF, STAT5B, TNFRSF10A, PRKCA*
Cyclins and Cell Cycle Regulation	11	1.13	2.49e-2	*MYT1, E2F4, HDAC2, SUV39H1, HDAC11, PPP2R5B, TGFB3, E2F5, GSK3B, CDKN1B, SIN3A*
**5** **hr**				
Calcium	31	−4.08	2.32e-4	*RAP2B, PRKACB, RAP2A, MYH10, Calm1 (includes others), CAMK4, NFATC3, PRKAG1, CABIN1, GRINA, EP300, HDAC6, CAMK2D, PPP3CB, TRPV6, PPP3R1, HDAC11, MAPK3, CAMK1G, RYR2, PPP3CC, PNCK, ATF2, GRIN3A, RCAN1, MICU1, CHRNB2, MEF2D, PRKAG2, RCAN3, CAMK2G*
Rac	19	−4.36	4.11e-3	*RELA, RPS6KB1, TIAM1, NRAS, PIK3R1, PIP4K2B, WASF1, PIK3R4, PLD1, LIMK1, MCF2L, ITGA3, CYFIP2, PAK3, MAPK3, CD44, PIK3CD, PAK7, SH3RF1*
NGF	19	−4.36	4.11e-3	*TP53, RELA, RPS6KB1, NRAS, PIK3R1, MAP3K4, PIK3R4, NGF, ATF2, EP300, SHC1, MAP3K12, IKBKB, PTPN11, MAPK3, NTRK1, PIK3CD, MAPK7, MAP3K2*
Rho Family GTPase	31	−4.16	3.06e-2	*RELA, ARHGEF7, PIK3R1, WASF3, PIP4K2B, PIK3R4, SLC9A1, LIMK1, ITGA3, GNAT1, RHOT1, MAPK3, ARHGEF11, ARHGEF12, CDH4, GNAI1, CDC42EP3, WASF1, PLD1, MAP3K12, GNAI3, FOS, CDH2, RHOV, PAK3, CDH8, PIK3CD, PAK7, ACTG1, ARHGEF9, ARHGEF10*
PTEN	19	2.36	1.67e-2	*RELA, RPS6KB1, NRAS, PIK3R1, ILK, FGFR2, OCRL, SHC1, IKBKB, ITGA3, CASP9, MAGI1, MAPK3, NTRK1, PIK3CD, CDKN1B, INSR, KDR, MAGI3*

**Table 5:** NF-κB Enriched Targets.

Regulation	Pattern	Time (Hr)	Transcripts		NF-κB Sites	
			ALL NF-κB	Prom	Total	Unique
Down	18/1	2	18.7	31.3	3.3	1.7
		5	17.2	27.2	2.1	1.5
Up		2	12.6	23.3	2.4	1.3
		5	18.3	30.3	2.5	1.3
Down	90/5	2	13.5	23.5	1.0	0.8
		5	10.7	18.5	0.7	0.6
Up		2	19.6	30.6	3.0	1.4
		5	25.1	36.5	4.5	2.5

NF-κB sites are enriched in transcripts following 5 hr stimulation by the 90/5 pattern. Transcripts up- and down-regulated by patterned stimulation for 2 hr and 5 hr by 18/1 or 90/5 stimulation patterns ( >1.4-fold and p < 0.05) were analyzed by DIRE. Transcripts containing NF-κB sites (NF-κB, NF-κB p50, and NF-κB p65) were filtered and summarized by transcript and TFBS. For NFK analysis by transcripts, the % of NFK-containing transcripts to total transcripts (Transcripts within proximal promoter or across intergenic, upstream, and ECRs) is given. For NF-κB TFBS analysis by site, unique sites or total # of observations were considered. In all 4 types of analysis, there was significant enrichment in NFK sites for 5 hr at 90/5 [Mean and 95% confidence interval for transcripts: 17.0 ± 3.2% (All NF-κB), 27.6 ± 3.9% (Promoter only); for NF-κB sites: 2.4 ± 0.8% (Total) and 1.4 ± 0.4% (Unique)].
