# Supplementary material for: Gene networks activated by specific patterns of action potentials in dorsal root ganglia neurons
Source: Sci Rep. 2017 Mar 3;7:43765. doi: 10.1038/srep43765 (PMC5335607; doi:10.1038/srep43765)
Supplement: Supplementary Material [file srep43765-s1.doc]

**Gene networks activated by specific patterns of action potentials in dorsal root ganglia neurons.**

Philip R. Lee, Jonathan E. Cohen, Dumitru A. Iacobas, Sanda Iacobas, and R. Douglas Fields

**
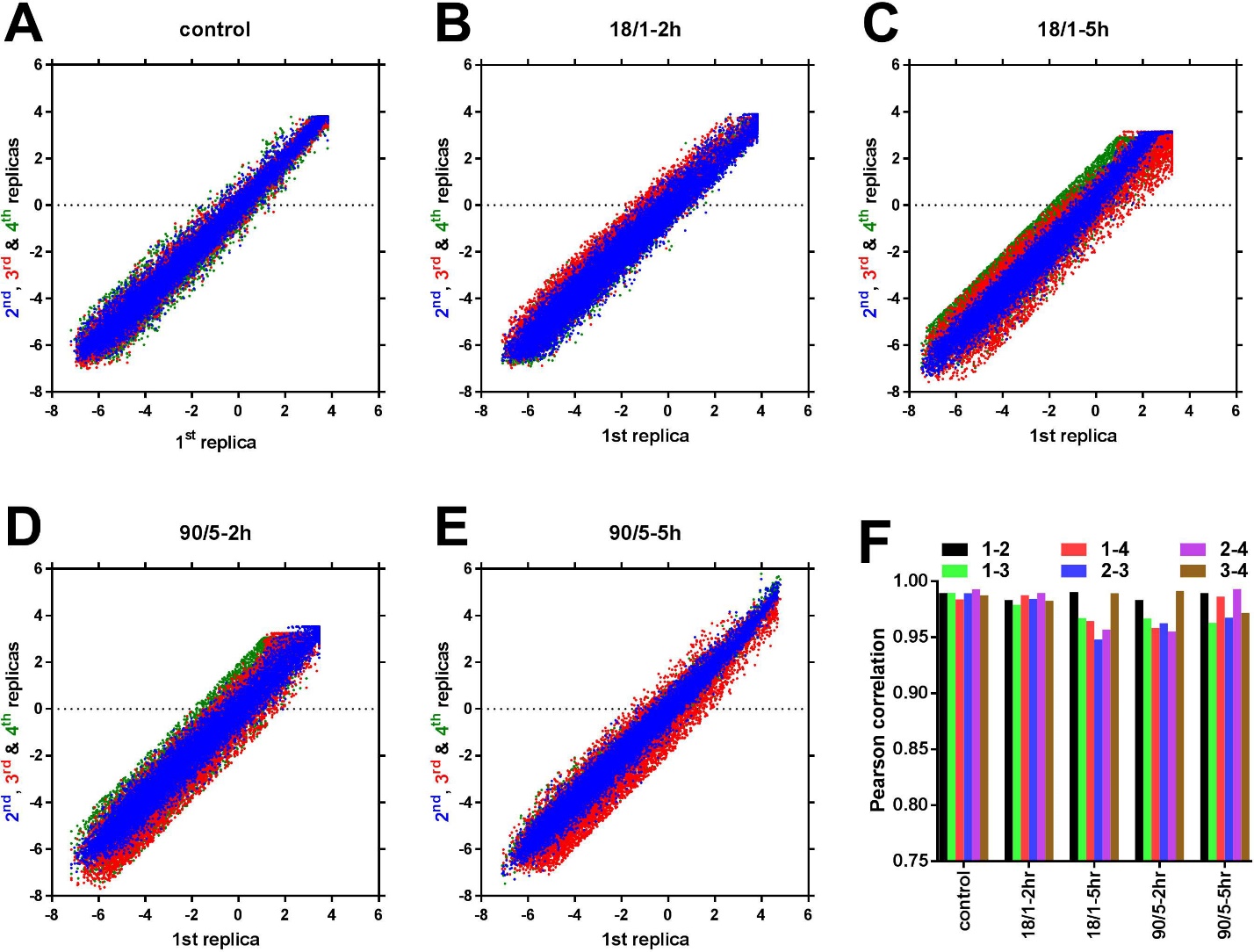
**

**Figure S1. Correlation of expression data among biological replicates of each condition. (A-E**) Log_2_ normalized expression levels of the 2^nd^, 3^rd^, and 4^th^, replicas were plotted against that of the 1^st^ replica. Correlations for pairs 2-3, 2-4 and 3-4 of replicates for each of the five conditions (not shown) were very similar as indicated by their Pearson coefficient (last three columns in each group from (F)) (**F**) Pearson product-moment correlation coefficient between the expression levels of all six pairs (1-2,1-3,1-4, 2-3, 2-4, 3-4) that can be formed with the four biological replicas in each condition. Note the high correlation between the gene expression levels in every pair (from 0.948 for pair 2-3, blue bar; in 18/1 for 5hr) to 0.993 for pair 2-4, purple bar; in 90/5 for 5hr). In F, each column represents the Pearson correlation between the expression values of >14k unigenes quantified in both indicated replicas (e.g 1 & 2 in the black column) of the respective condition. The high correlation between microarray samples demonstrates the reproducibility and sensitivity of the Agilent microarray platform

**
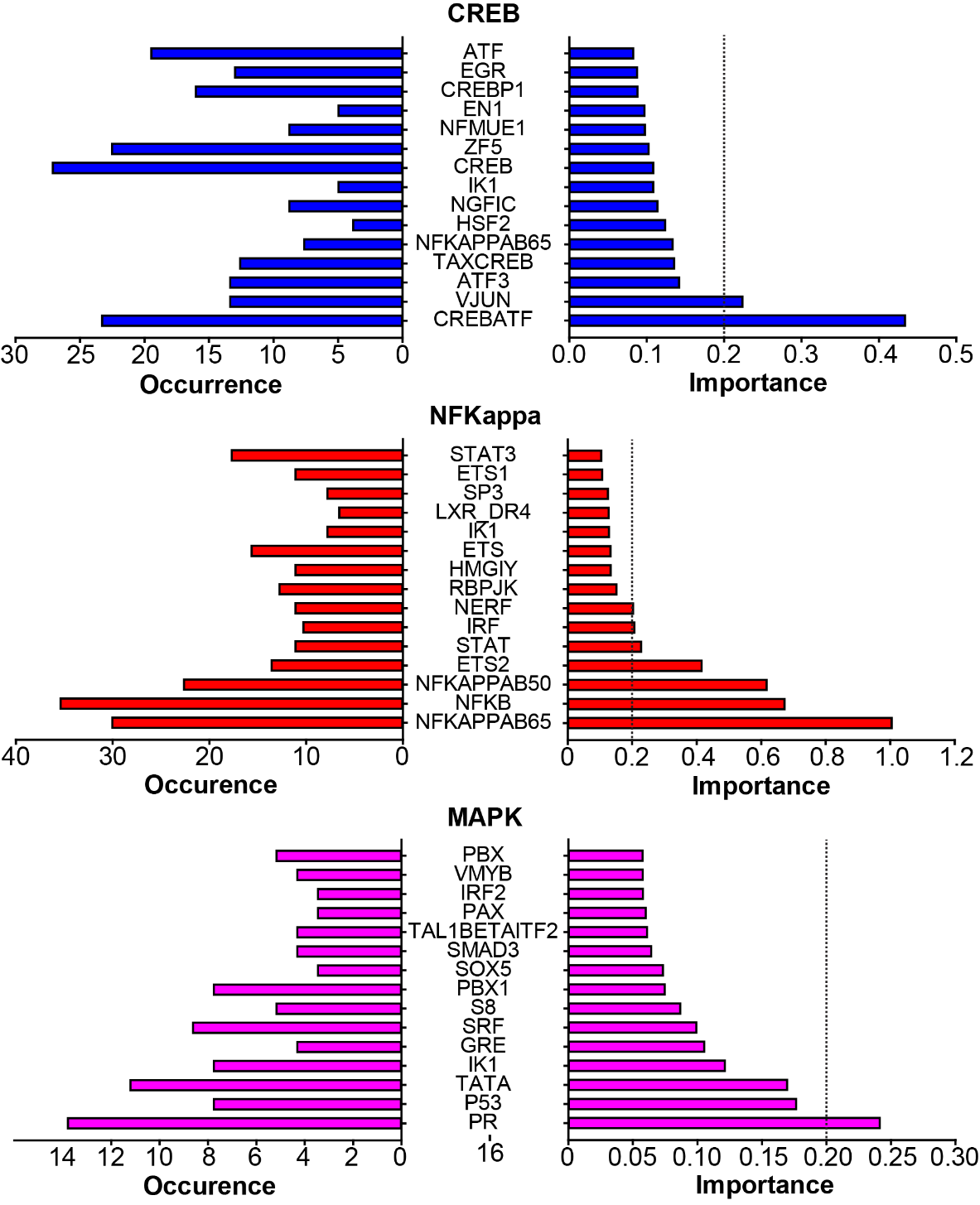
**

**Figure S2. Enrichment of TFBS identified in CREB, NF-κB** **and MAPK gene sets.** Top 10 candidate cis-regulatory elements identified by DIRE^1^ in selected datasets corresponding to CREB (significantly up-regulated transcripts)^2^, NF-κB (identified targets of NF-κB signaling) (http://www.bu.edu/nf-kb/gene-resources/target-genes/), and MAPK regulated transcripts (down-regulated transcripts in ERK1/2 cKO mice)^3^. For each TF enriched dataset, % occurrence and importance factor are given for the top 10 enriched sites. DIRE analysis was performed using a background get of 5,000 randomly selected genes and top 3 ECRs + promoter ECRs.

**Table S1. mRNA regulation of immediate early genes (IEG) and NF-κB genes, measured by RT-PCR.** NPAS4 (Neuronal PAS Domain Protein 4) and EGR1 (KROX-24/zif-268) were two of the most highly regulated transcripts identified by microarray for all conditions and were selected as representative IEGs. PIM1, NFKBIE, and BCOR containing from 1 to 6 unique sites, were selected as representative NF-κB -responsive genes.

| **stimulation /time** | **gene** | **mean ± SEM** | **N** | **t-value** | **P-value** | **significance** | **# NF-κB sites** |
| --- | --- | --- | --- | --- | --- | --- | --- |
| 90/5 @ 5hr | NPAS4 | 99.9 ± 17.9 | 3 | 5.5 | 0.016 | * | - |
| 90/5 @ 5hr | EGR1 | 20.0 ± 2.4 | 3 | 7.8 | 0.008 | ** | - |
| 90/5 @ 5hr | NFKBIE | 2.5 ± 0.4 | 4 | 3.6 | 0.019 | * | 4 |
| 90/5 @ 5hr | PIM1 | 2.1 ± 0.5 | 6 | 2.1 | 0.043 | * | 6 |
| 90/5 @ 5hr | BCOR | 2.0 ± 0.1 | 3 | 13.3 | 0.003 | *** | 1 |
|  |  |  |  |  |  |  |  |
| 18/1 @ 5hr | NPAS4 | 40.4 ± 12.1 | 3 | 3.3 | 0.041 | * | - |
| 18/1 @ 5hr | EGR1 | 88.9 ± 26.6 | 3 | 3.3 | 0.040 | * | - |
| 18/1 @ 5hr | NFKBIE | 0.9 ± 0.3 | 3 | -0.4 | 0.761 |  | 4 |
| 18/1 @ 5hr | PIM1 | 1.0 ± 0.2 | 3 | 0.2 | 0.885 |  | 6 |
| 18/1 @ 5hr | BCOR | 1.2 ± 0.1 | 3 | 1.5 | 0.282 |  | 1 |

*p ≤ 0.05, **p < 0.01, and ***p < 0.005; t-test, comparing to corresponding control, unstimulated cultures. Mean and SEM values correspond to normalized fold changes relative to GAPDH. N = 1 represents pooling of 4 culture dishes (8 separate compartments) combined for analysis of transcript mRNA. # NF-κB sites corresponds to unique NF-κB sites from DIRE analysis.

**Table S2. Transcription factor signatures by gene set enrichment analysis.** Filtered transcriptome data (N=4 replicates per condition) was analyzed by GSEA using the molecular signatures database (MSigDB) C3, motif gene sets, comprised of 836 gene sets corresponding to miRNA targets (221) and transcription factor targets (615). Gene set enrichment statistics are given, as the normalized enrichment score (NES)^4^ defined as the enrichment score/mean enrichment score against all permutations in the dataset) and the false discovery rate (FDR) in parenthesis. TFBS were considered when the FDR <10% (FDR stringency was adjusted in order to discover gene set signatures as described in the GSEA methods).

|  |  | **18/1** | | **90/5** | |
| --- | --- | --- | --- | --- | --- |
|  | **TFBS** | **2HR** | **5HR** | **2HR** | **5HR** |
| **SRF** |  |  |  |  |  |
|  | V$SRF_C | **2.28 (0.02)** |  | 1.82 (0.39) | **1.96 (0.07)** |
|  | V$SRF_Q4 | **2.27 (0.01)** |  | 1.68 (0.26) | **1.80 (0.09)** |
|  | CCAWWNAAGG | **1.96 (0.03)** |  | 1.55 (0.41) | 1.48 (0.26) |
|  | V$SRF_Q6 | **1.90 (0.05)** |  |  | 1.63 (0.12) |
| **CREB** |  |  |  |  |  |
|  | V$ATF6_01 | **2.15 (0.02)** |  |  |  |
|  | V$CREBP1_Q2 | **2.06 (0.03)** |  |  |  |
|  | V$CREB_Q2 | **1.88 (0.05)** |  |  |  |
|  | V$ATF_B | **1.84 (0.05)** |  |  |  |
|  | V$CEBPDELTA_Q6 | **1.83 (0.05)** |  |  |  |
|  |  |  |  |  |  |
| **NFK** | V$NFKB_C |  |  |  | **1.95 (0.04)** |
|  | V$NFKAPP65_01 |  |  |  | **1.75 (0.08)** |
|  | V$NFKB_Q6_01 |  |  |  | **1.75 (0.08)** |
|  | V$CREL_01 |  |  |  | 1.64 (0.12) |
|  | V$NFKAPPAB_01 |  |  |  | 1.54 (0.20) |

**Table S3. DIRE Prediction of Transcription Factor Binding Sites.** % occurrence and importance factor for 311 predicted TFBS identified by DIRE analysis. Results summarized in Fig, 4 and Fig. 5 are given for both electrical stimulation pattern (18/1 and 90/5) and duration (2 hr and 5 hr). TFBS were identified in evolutionary conserved regions (ECRs), upstream promoters, and 5’-UTRs. As detailed in the Methods, default parameters e.g. a large random set of transcripts (5,000), does not accurately represent tissue-specific expression of transcripts and corresponding TFBS present in DRG neurons and would skew the predicted importance factor. TF weighting to calculate importance factor was performed by applying a background gene set of moderately expressed transcripts not regulated by either pattern at 2 hr and 5 hr.

**REFERENCES**

1. Gotea, V. & Ovcharenko, I. DiRE: identifying distant regulatory elements of co-expressed genes. *Nucleic Acids Res*. Jul **1;36** (Web Server issue):W133-9 (2008).
2. Benito, E., Valor, L.M., Jimenez-Minchan, M., Huber, W. & Barco, A. cAMP response element-binding protein is a primary hub of activity-driven neuronal gene expression. *J Neurosci* **31**(50):18237–18250 (2011).
3. Newbern, J.M. *et al.* Specific functions for ERK/MAPK signaling during PNS development. *Neuron*. Jan 13;**69(1):**91-105 (2011).
4. Subramanian, A. *et al.* Gene set enrichment analysis: a knowledge-based approach for interpreting genome-wide expression profiles. *Proc Natl Acad Sci U S A*. Oct 25;**102(43)**:15545-50 (2005).
